# Hemolytic-uremic syndrome: 24 years’ experience of a pediatric nephrology unit

**DOI:** 10.1590/2175-8239-JBN-2021-0206

**Published:** 2022-04-04

**Authors:** Ana Sofia Vilardouro, Joana Cachão, Márcia Rodrigues, Filipa Durão, Patrícia Costa-Reis, Ana Rita Sandes, José Esteves da Silva, Leonor Boto, Rosário Stone

**Affiliations:** 1 Hospital de Santa Maria, Centro Hospitalar Universitário Lisboa Norte, Unidade de Nefrologia Pediátrica e Transplante Renal, Departamento de Pediatria, Lisboa, Portugal.; 2 Hospital de São Bernardo, Centro Hospitalar de Setúbal, Serviço de Pediatria, Setúbal, Portugal.; 3 Hospital de Santa Maria, Centro Hospitalar Universitário Lisboa Norte, Serviço de Genética, Departamento de Pediatria, Lisboa, Portugal.; 4 Universidade de Lisboa, Faculdade de Medicina, Lisboa, Portugal.; 5 Hospital de Santa Maria, Centro Hospitalar Universitário Lisboa Norte, Unidade de Cuidados Intensivos Pediátricos, Departamento de Pediatria, Lisboa, Portugal.

**Keywords:** Hemolytic-Uremic Syndrome, Thrombotic Microangiopathies, Eculizumab, Kidney Transplantation, Síndrome Hemolítico-Urêmica, Microangiopatias Trombóticas, Eculizumab, Transplante de Rim

## Abstract

**Introduction:**

A better understanding of hemolytic-uremic syndrome (HUS) pathophysiology significantly changed its treatment and prognosis. The aim of this study is to characterize the clinical features, severity, management, and outcomes of HUS patients.

**Materials and Methods:**

Retrospective study of HUS patients admitted to a Pediatric Nephrology Unit between 1996 and 2020. Demographic and clinical data regarding etiology, severity, treatment strategies, and patient outcome were collected.

**Results:**

Twenty-nine patients with HUS were admitted to our unit, but four were excluded. Median age at diagnosis was two years (2 months - 17 years). Clinical manifestations included diarrhea, vomiting, oliguria, hypertension, and fever. During the acute phase, 14 patients (56%) required renal replacement therapy. Infectious etiology was identified in seven patients (five *Escherichia coli* and two *Streptococcus pneumoniae*). Since 2015, 2/7 patients were diagnosed with complement pathway dysregulation HUS and there were no cases of infectious etiology detected. Six of these patients received eculizumab. The global median follow-up was 6.5 years [3 months-19.8 years]. One patient died, seven had chronic kidney disease, four of whom underwent kidney transplantation, one relapsed, and seven had no sequelae.

**Conclusion:**

These results reflect the lack of infectious outbreaks in Portugal and the improvement on etiological identification since genetic testing was introduced. The majority of patients developed sequels and mortality was similar to that of other countries. HUS patients should be managed in centers with intensive care and pediatric nephrology with capacity for diagnosis, etiological investigation, and adequate treatment. Long-term follow-up is essential.

## Resumo

**Introdução:**

Um melhor entendimento da fisiopatologia da síndrome hemolítico-urêmica (SHU) mudou significativamente seu tratamento e prognóstico. Este estudo teve como objetivo caracterizar condições clínicas, gravidade, manejo e desfechos de pacientes com SHU.

**Materiais e Métodos:**

Estudo retrospectivo de pacientes com SHU admitidos numa Unidade de Nefrologia Pediátrica entre 1996-2020. Foram coletados dados demográficos e clínicos sobre etiologia, gravidade, estratégias de tratamento, desfechos de pacientes.

**Resultados:**

29 pacientes com SHU foram admitidos em nossa unidade, mas quatro foram excluídos. A idade mediana ao diagnóstico foi dois anos (2 meses-17 anos). Manifestações clínicas incluíram diarreia, vômitos, oligúria, hipertensão e febre. Durante a fase aguda, 14 pacientes (56%) necessitaram de terapia renal substitutiva. Identificou-se a etiologia infecciosa em sete pacientes (cinco *Escherichia coli*; dois *Streptococcus pneumoniae*). Desde 2015, 2/7 pacientes foram diagnosticados com SHU por desregulação da via do complemento e não foram detectados casos de etiologia infecciosa. Seis desses pacientes receberam eculizumab. A mediana global de acompanhamento foi 6,5 anos [3 meses-19,8 anos]. Um paciente faleceu, sete apresentaram doença renal crônica, sendo quatro submetidos a transplante renal, uma recidiva e sete sem sequelas.

**Conclusão:**

Estes resultados refletem a ausência de surtos infecciosos em Portugal e a melhoria na identificação etiológica desde que os testes genéticos foram introduzidos. A maioria dos pacientes desenvolveu sequelas e a mortalidade foi semelhante à de outros países. Pacientes com SHU devem ser manejados em centros com cuidados intensivos e nefrologia pediátrica com capacidade para diagnóstico, investigação etiológica e tratamento adequado. O acompanhamento alongo prazo é essencial.

## Introduction

Hemolytic-uremic syndrome (HUS) is a thrombotic microangiopathy characterized by the classic triad: hemolytic anemia, thrombocytopenia and acute kidney injury. In the last decade, there was great progress on the understanding of HUS etiology and pathophysiology. The role of complement regulation was unveiled and a new classification of HUS based on its pathogenic mechanisms, instead of the traditional classification of diarrhea positive HUS (D+HUS) and diarrhea negative HUS (D-HUS), was proposed. The 2016 International Hemolytic Uremic Syndrome Group classification is organized considering HUS etiology as: 1) infection-induced HUS (Shiga toxin producing *Escherichia coli, Streptococcus pneumoniae,* Influenza A, human immunodeficiency virus); 2) HUS with coexisting diseases or conditions (bone marrow or solid organ transplantation, systemic malignancies, autoimmune conditions, drugs, malignant hypertension); 3) HUS due to cobalamin C disorder; and 4) HUS due to alternative complement pathway dysregulation and mutation in diacylglycerol kinase ε (*DGKE*) gene^1-4^.

HUS associated with Shiga-toxin producing *Escherichia coli* (STEC) is the most frequent cause, representing 85 to 90% of all pediatric cases^5^. Invasive infections by *Streptococcus pneumoniae* account for approximately 5% of cases and genetic mutations associated with dysregulation of the alternative complement pathway account for 5-10% of patients^5^.

While there are multiple triggers leading to HUS, all of them are responsible for the same pattern of endothelial cell damage in the microvasculature of multiple organs, mainly the kidney and the brain, and similar clinical and biological abnormalities^2^.

HUS is rare, but it can be a severe illness with important morbidity and mortality.

Our clinical practice follows the recommendations of the international consensus by Loirat C *et al*.^3^ The introduction of eculizumab, the first drug to effectively block complement activation, has greatly changed the treatment and outcome of patients with HUS due to alternative complement pathway dysregulation. An early recognition of the disease’s presentation and initiation of treatment is vital to minimize organ injury.

The aim of this study was to characterize the clinical features, flares, etiology, management, morbidity, and mortality of HUS in pediatric patients admitted to our Unit over the past 24 years.

## Materials and Methods

This was a retrospective and descriptive study of all patients admitted with HUS diagnosis in the Pediatric Nephrology Unit of a Portuguese tertiary hospital during the 24-year-period between January 1996 and March 2020. All medical files were reviewed and demographic, clinical, and laboratory data concerning etiology, severity, management and patient outcome were collected. Patients with no data available were excluded from the study. Clinical data on the last clinical visit was obtained. Minor sequelae were defined as the presence of high blood pressure (HBP) and/or non-nephrotic proteinuria with glomerular filtration rate (GFR) ≥ 90 mL/min/1.73m^2^. Chronic kidney disease (CKD) was defined by a GFR lower than 90 mL/min/1.73m^2^. GFR was estimated using the Schwartz Equation (mL/min/1.73m^2^) = 0.413 x height (cm)/serum creatinine (mg/dL). STEC was identified by serological and/or microbiological studies of stool samples and polymerase chain reaction (PCR) after 2012. *Streptococcus pneumoniae* was identified using microbiological studies or urine immunochromatography.

Genetic tests have been performed since 2015 in the Laboratory of Molecular Hematology of Coimbra University Hospital Center and in two other private laboratories of molecular diagnostic testing. There was no uniformity on genetic testing nor on the gene panels used. The variants were reported according to the American College of Medical Genetics and Genomics (ACMG) guidelines.

In our institution, eculizumab was available since 2015 and was started in the first 24-48 hours if severe HUS and/or a high index of suspicion of an alternative complement pathway dysregulation. Patients treated with eculizumab received the quadrivalent meningococcal conjugate, the serogroup B meningococcal vaccines, and prophylactic antibiotics. Genetic testing and eculizumab were covered by the Portuguese National Health Service.

Patients were divided into two historical cohorts for better characterization and evaluation of the follow-up period. Group A included patients admitted before 2015, when genetic testing and eculizumab were not available in our institution, and Group B included patients admitted since 2015. One patient was included in both groups because he had a first episode of HUS before 2015 and relapsed in the second time period when the genetic testing was performed.

The study was submitted to and approved by the Ethical Committee of our institution.

## Results

### Epidemiological Data

**Figure 1:**
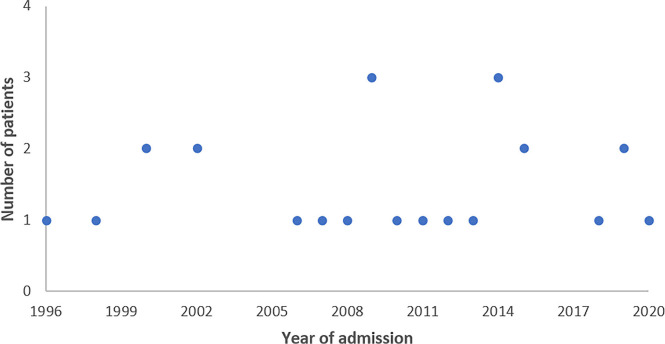
Study flowchart.

**Figure 2:**
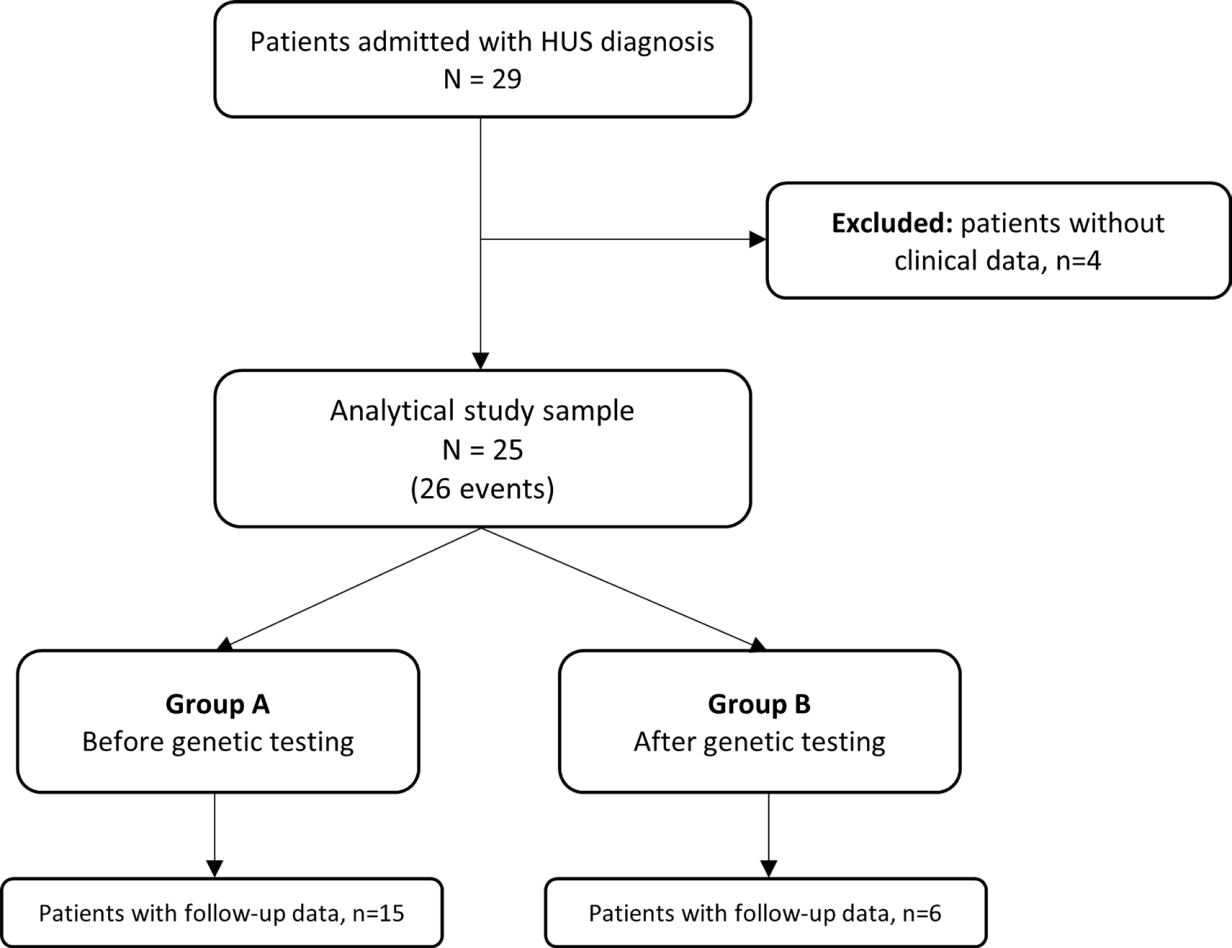
Scatter plot of cases over time.

During the study period there were 29 patients with HUS admitted to the Pediatric Intensive Care Unit of our hospital and followed by our Pediatric Nephrology Unit (Figure 1). Four patients were excluded due to lack of data. Twenty-five patients with 26 events met the inclusion criteria, 64% of whom were male, with a median age at diagnosis of 2 years (2 months-17 years). Group A had 19 individuals and Group B had 7 patients (Figure 2). None of the children had a family history of HUS. The median length of stay at the hospital was 28 days (4-191 days).

### Clinical and Laboratory Presentation

As shown in Table 1, the most frequent clinical manifestations were diarrhea (76%), vomiting (68%), fever (48%), and edema (32%). Only three patients had bloody diarrhea. During hospital stay, 96% developed HBP, 72% developed oligoanuria, and 16% had neurological impairment (n=2 seizures, n=2 somnolence). One patient died during the acute phase of the disease due to neurologic involvement, which was present from admission (no etiology was identified). Laboratory findings on admission were: average hemoglobin of 6.3 ± 1.35 g/dL [3.2-9.0 g/dL]; thrombocytopenia in all but one patient, with a mean platelet count of 58,188/µL ± 46,638/µL [11,000-193,000/µL]; mean creatinine of 5.0 ± 3.8 mg/dL [0.7-15.7 mg/dL], corresponding to an average GFR (N=14) of 14.5 ± 10.5 mL/min/1.73m^2^ [3.6-35.2mL/min/1.73m^2^].

**Table 1:** Patients’ clinical presentation, therapy, and support measures (N=25)

		n	%
Demographic data	Number of patients	25	-
	Number of events	26	-
	Males	17	68%
	Median age (years) [minimum-maximum]	2 [2 months - 17 years]	-
Clinical presentation	High blood pressure	24	96%
	Diarrhea	19	76%
	(sanguinolent diarrhea)	(3)	(16%)
	Oligoanuria	18	72%
	Vomiting	17	68%
	Fever	12	48%
	Edema	8	32%
	Abdominal pain	3	12%
	Somnolence	2	8%
	Seizures	2	8%
	Hematuria	2	4%
Therapy and support measures	Renal replacement therapy:	14	56%
	- PD	7	50%
	- CVVHDF	3	22%
	- PD + CVVHDF	3	22%
	- CVVHDF + HD	1	7%
	Plasmapheresis	2	8%
	Erythrocyte transfusion	15	60%
	Mechanical ventilation	5	20%
	Specific therapy:		
	- Eculizumab	6	24%*

CVVHDF: Continuous venovenous hemodiafiltration; HD: Hemodialysis; PD: Peritoneal dialysis.

* Corresponding to 86% of the patients treated since 2015.

### Treatment

During the acute phase, 14 patients (56%) required renal replacement therapy (Table 1), 11 patients from group A and 3 patients from group B. Seven (50%) had peritoneal dialysis, three (22%) had continuous venovenous hemodiafiltration (CVVHDF), three (22%) had both therapies, and one patient (7%) required CVVHDF and hemodialysis. Median duration of peritoneal dialysis was 22 days [6-180 days] and of CVVHDF was five days [3-19 days].

Eculizumab was administered to six of the seven patients treated since 2015. Three of the patients who received eculizumab needed renal replacement therapy during acute phase. In contrast, eleven (58%) of the patients of Group A needed renal replacement therapy, representing 86% (n=6) of the patients with infectious etiology.

As for other treatments, plasmapheresis was used in two (8%) patients (one had anti-factor H antibodies and the other patient had no identifiable etiology; one of these patients was from Group A and the other from Group B) and 15 (60%) required erythrocyte transfusion. None of the patients required cardiovascular support. Mechanical ventilation was required in five patients (20%), all belonging to Group A.

### Etiologic Investigation

The etiology was identified in nine patients (36%) (Tables 2 and 3). Seven patients had an infection: five with a Shiga-toxin producing *E. coli* and two with *S. pneumoniae* (one identified in pleural effusion and the other with S. *pneumoniae* antigens detected in urine).

Six of the seven patients from group B had genetic analysis performed and five of these patients had an identifiable variant. Two patients were diagnosed as having a complement dysregulation HUS: one patient with a complete homozygous deletion of complement factor H-related protein 1 (*CFHR1*) and complement factor H-related protein 3 (*CFHR3*), who had anti-factor H antibodies production, and one patient with a likely homozygous pathogenic variant in *C3* gene. The other three patients harbored variants of unknown significance (VUS): a complete homozygous deletion of *CFHR1* and *CFHR3* without anti-factor H antibodies production, a heterozygous mutation in genes encoding complement factor I (*CFI*), and a heterozygous variant in the complement regulatory gene factor H (*CFH*) (Table 2).

**Table 2:** Etiological study of the patients

	Genes/ variants	Classification	n
Complement dysregulation (genetic analysis performed in 6 patients)	*C*3: c.193A>C, p.(Lys65Gln) in homozygosity	Likely pathogenic variant	1
	*CFI*: c.530T>A, p.(Asn177Ile) in heterozygosity (plus 2 risk haplotypes in *CFH* and in *MCP*)	VUS	1
	*CFH*: c.3653G>T, p.(Cys1218Phe) in heterozygosity		1
	Complete homozygous deletion of *CFHR1* and *CFHR3* (with anti-factor H antibodies production)		1
	Complete homozygous deletion of *CFHR1* and *CFHR3* (without anti-factor H antibodies production)		1
			
Specific infectious agent (infectious etiological search performed in 16 patients)	*E. coli*		5
	*S. pneumoniae*		2

CFI: complement factor I; CFH: complement factor H; CFHR1: complement factor H-related protein 1; CFHR3: complement factor H-related protein 3; MCP: membrane cofactor protein; VUS: variant of unknown significance.

None of the patients with neurologic involvement (n=4) had an identifiable etiology, but only two of them had a complete complement dysregulation investigation performed. One of these patients had a heterozygous mutation in *CFH* gene, which is considered a VUS.

### Follow-up

One patient died during the acute phase of the disease. There were no deaths to report during the follow-up period (24/24 patients). There was data regarding the last clinical visit for 20 patients (Table 3). Median follow-up duration was 6.5 years (3 months-19.8 years).

**Table 3:** Characterization of the patients considering two historical cohorts - before genetic testing versus after genetic testing era - and their follow-up

	Group A Before genetic testing	Group B After genetic testing
Number of patients with follow-up data after discharge	15* ^§^	6^† §^
Number of patients that received eculizumab	NA	6
Number of genetic tests performed	NA	6
Identified etiology:		
- Shiga-toxin producing *E. Coli*	5	0
- *S. pneumoniae*	2	0
- Anti-factor H antibodies	0	1
- Genetic variants pathogenic/likely pathogenic in complement-related genes	NA	1
- No etiology identified	12	5
Renal replacement therapy (PD or HD at any time during follow-up period)	5	1
Number of deaths	1	0
No sequelae	5	2
Hematologic remission	15	6
Neurologic impairment	0	0
Kidney sequelae	6	2^§^
- Minor sequelae		
HBP only	1	2
HBP + non-nephrotic proteinuria	3	0
- Chronic kidney disease		
G2	2	-
G3a	4	1
G4	-	1
Relapses	1	0
Kidney transplant	4	0

§ One of these patients only had Eculizumab on the relapse episode, as it was not available on the first episode.

* Three patients had no follow-up data (considering our Unit database and national digital records, they were never under dialysis treatment) and one patient died.

† One patient had no follow-up data (considering our Unit database and national digital records, he was never under dialysis treatment).

HBP: High blood pressure; CKD: Chronic kidney disease; HD: Hemodialysis; NA: Not applicable; PD: Peritoneal dialysis.

In Group A (N=19), there was follow-up data of 15 patients, with a median duration of 10.5 years (4.8-19.8 years). At the last clinical visit, 33% (n=5) patients had no renal sequelae, 27% (n=4) had minor sequelae (three patients with HBP and non-nephrotic proteinuria, one with HBP only), 40% (n=6) had CKD, and none of the patients had neurologic sequelae. Only one patient from this cohort relapsed (7 and 9 years after diagnosis). This patient (also included in group B) had a heterozygous mutation in *CFI* combined with risk haplotypes in *CFH* and *MCP*. Only this patient underwent kidney biopsy, which disclosed a membranoproliferative glomerulonephritis type I. From this group, 4/15 (27%) patients, kept renal replacement therapy dependence after the acute phase and all of them underwent kidney transplantation (KT). Among the patients with CKD, 33% (n=2) are receiving conservative treatment and 67% (n=4) started peritoneal dialysis and underwent KT. From the latter group, three patients had a STEC-HUS and one had *S. pneumoniae* infection. Average time between diagnosis and KT was 6.4 ± 2.7 years [3.5-10 years]. There was no recurrence of disease on renal graft in any of the patients. All patients achieved hematologic remission.

In Group B (N=7), we had follow-up data of six patients, with a median duration of 11 months (6.2 months-4.2 years). At the last follow-up evaluation, 33% had no sequelae (n=2; one of these patients had anti-factor H antibodies and eculizumab was not administered), 67% had HBP (n=4), 33% had CKD (n=2), and none of the patients underwent KT. From this group, one patient (17%) kept renal replacement therapy dependence (hemodialysis) after the acute phase, but the patient is currently under conservative treatment. Among the group with CKD (N=2), both patients are under conservative therapy. There were no adverse drug reactions during the acute phase. The only adverse reaction associated to eculizumab use occurred during the follow-up period, characterized by edema of lower limbs during its administration, which improved with antihistamines and a slower infusion. There were no HUS relapses in these patients.

In both groups, oligoanuria was part of the clinical presentation in six out of seven patients (86%) that developed CKD and in nine of thirteen patients (69%) that did not develop CKD. All patients achieved hematologic remission.

In the group of patients with infection-induced HUS (N=7), all cases were diagnosed before 2015, all patients required renal replacement therapy during the acute phase of disease, all had sequelae, and four out of seven patients with CKD underwent a KT.

Of the two patients with complement dysregulation HUS, one developed HBP, and none developed proteinuria or CKD. The patient that developed HBP was treated with eculizumab.

There were two patients that continued receiving eculizumab periodically after the acute episode (the patient with a homozygous C3 variant and the patient with a complete homozygous deletion of *CFHR1* and *CFHR3* with no anti-factor H antibodies production, but with complement consumption) with good response. The first patient mentioned is currently receiving eculizumab for 30 months and the other one for 6 months.

## Discussion

During the 24 year-period of our study, there were 29 cases of HUS referred to our hospital. In Portugal, the incidence of HUS is unknown. However, our Pediatric Intensive Care Unit (PICU) has the highest admission rate in the country, which lead us to think that the low number of cases throughout the years is probably because of the low incidence of this disease^6^. A Norwegian study reports an annual incidence of 0.5 cases per 100,000 children^7^. The overall incidence of HUS in United Kingdom and Ireland is 0.71 per 100,000 children under 16 years of age, and incidence is similar across Europe, Australia, and North America^8^.

The classic triad combining thrombocytopenia, hemolytic anemia, and acute kidney injury is the typical hallmark of this condition^3^. Only one patient did not present with thrombocytopenia, which may be absent at presentation in 15-20% of patients.^2^ The median age of HUS in our cohort was two years, in accordance to other studies that indicate that HUS is more frequent in children of preschool age^9^. However, this is mainly true for infection-induced HUS, while the onset of HUS associated with complement dysregulation occurs in children almost as frequently as in adults.

In our study, mortality was 4%, which corresponds to the rates in developed countries, where HUS mortality is below 5%^9-11^.

In most cases, HUS presents abruptly and with nonspecific clinical symptoms of HUS, which include reduced urine output and edema, and are usually related to a triggering infectious event. Some clinical manifestations may arise suspicion on the underlying etiology, but in the great majority of cases the clinical picture is not sufficient to identify the condition^2,5^.

STEC-HUS frequently follows prodromal bloody diarrhea, which was present in only one of the five cases with confirmed STEC infections^2^. All the remaining cases had non-bloody diarrhea. Extrarenal manifestations are thought to occur due to multisystem thrombotic microangiopathy. Neurologic involvement is the most common life-threatening extrarenal manifestation (3-26%)^2,5^. In complement dysregulation associated HUS, extrarenal manifestations occur in approximately 20% of cases, with neurologic involvement being the most common, estimated at 10%. Nevertheless, in our cohort study, none of the four patients with CNS involvement had an identified etiology. However, only two of them had genetic testing performed and while one of them had a heterozygous mutation in the *CFH* gene, this is considered a VUS. The development of HBP in the acute phase is frequent, as shown see in our case series (96%)^2,5,12-14^.

Only 36% of the patients had an identifiable etiology, being STEC-HUS the most common infection-induced HUS (71%) and representing 56% of all cases with identifiable etiology.

On the other hand, 83% of the patients with a genetic test performed had an identified variant in complement regulatory genes, although only one was considered a pathogenic variant (homozygous C3 variant). Recent functional studies suggest that the variant detected in *CFI* gene [c.530T>A, p.(Asn177Ile)] is a causative variant, allowing its classification as a likely pathogenic variant instead of a VUS^15^. Moreover, although a complete homozygous deletion of *CFHR1* and *CFHR3* is not by itself considered pathogenic, it can be considered a risk factor for the development of HUS, and in the majority of cases it is associated with production of anti-factor H antibodies, as occurred in one of our patients^16,17^.

Other cohorts have described STEC as causing 85-95% of cases in children and genetic dysregulation of the alternative complement pathway as causing 5-10% of cases^5^. The low number of cases with infectious etiology in our sample is probably due in part to limitations in access to techniques of STEC identification, namely the unavailability of polymerase chain reaction techniques for some patients. In addition, these low numbers also reflect the lack of infectious STEC outbreaks in our country, making HUS an even rarer disease.

In our cohort, the seven patients with HUS of identified infectious etiology all needed renal replacement therapy and presented sequelae. All of these patients were admitted before genetic testing and eculizumab were available. For this reason, some of them could share genetic etiology and therefore benefit from treatment with eculizumab, which could lead to a better evolution and disease prognosis. Additionally, it is now known that the Shiga-toxin contributes not only to a proinflammatory and prothrombotic status, but it also induces complement activation, suggesting that eculizumab may be useful for patients with STEC-HUS^5,18-21^. Another factor to consider is that in some patients it was not possible to perform a complete etiology investigation because of the methodological deficiencies mentioned earlier, so some patients with infectious etiology and good evolution may have been missed. Finally, considering that we are a tertiary care hospital, the most serious cases are referred to our hospital.

In our cohort, there was no recurrence of disease after KT^13,22,23^. We emphasize that all the kidney transplanted patients had an infectious etiology identified for HUS, however a complement dysregulation study was not completed for all patients, which could stand for a less likely recurrence.

Before the use of eculizumab, outcomes of complement dysregulation-associated HUS were quite poor^24^. Although none of the patients that received eculizumab in our study required KT so far, we cannot take any conclusions, as this is a very small series of cases.

Genetic testing for complement pathway study was not available before 2015, making it difficult to understand if the poor prognosis of cases without a known etiology was related to complement dysregulation.

All patients were admitted to the Pediatric Intensive Care Unit, given the possible complications associated with this entity. Timely management of HUS is the most important factor in these cases, requiring prompt transfer to a referral treatment center to provide the optimal treatment^8^.

Renal replacement therapy is needed in 50-70% of cases in the acute phase of HUS, as seen in our cohort (56%), but there is no clear benefit for a specific type of renal replacement therapy, therefore it should be chosen according to the center experience and the patient’s condition^5,12,25^.

In this cohort, most patients in both groups had sequelae (67%) before and after eculizumab availability, a proportion that is twice as high as that described in the literature. Available data reveals that most patients recover kidney function, but up to about 25% evolve with sequelae, most frequently hypertension, proteinuria, and CKD^26,27^. Furthermore, six of seven patients (86%) that developed CKD had oligoanuria as clinical presentation, reflecting the poorer prognosis of these patients^28^.

HUS patients must have a long-term follow-up by a pediatric nephrologist, since CKD can occur years after the renal insult^29^. Children have a large renal functional reserve and the non-affected nephrons can compensate for the ones affected by the insult. It is highly recommended to follow these patients carefully in order to diagnose minor sequelae and implement renal protective strategies.

Genetic testing helped to increase the identification of HUS etiology. This has an impact on patient prognosis, as it can help to identify a greater proportion of patients with complement pathway dysregulation HUS who may benefit from a prolonged treatment with eculizumab^1^. Nevertheless, we must discriminate between the pathogenic and the likely pathogenic variants, as not all of are known to be responsible for the development of HUS. In our study, only one of the five identified variants was pathogenic.

## Conclusion

Over the last two decades there has been a significant progress in the diagnostic and therapeutic approach of patients with HUS, which makes their comparison difficult. Nonetheless, this study is relevant because it presents data from a representative series of patients throughout a long follow-up period.

This series confirms the high morbidity of HUS. Due to the severity of this disease, illustrated by the results of this cohort, it is essential to ensure early recognition of these patients and their transfer to a center with pediatric intensive care and nephrology units capable of performing a detailed etiological investigation, renal replacement therapy as necessary, and eventually prompt treatment with eculizumab. Long-term follow-up is required, even in patients who seem to recover completely from the acute phase. The influence that many genetic variants have on HUS development is still an open issue and further understanding in this area is needed.

### Limitations of the study

This study is unique in that it had a long-term follow-up of 24 years and describes two different eras of HUS treatment - before and after eculizumab use. However, it has some limitations, most notably the lack of data of some of the early patients admitted to our unit, which is related to the retrospective nature of the study. Also, genetic testing and extended complement studies have only been available since 2015, which may have led to some diagnosis of HUS being missed due to alternative complement pathway dysregulation.
